# Study toward a More Reliable Approach to Elucidate the Lignin Structure–Property–Performance Correlation

**DOI:** 10.1021/acs.biomac.3c00906

**Published:** 2023-12-19

**Authors:** Daryna Diment, Oleg Tkachenko, Philipp Schlee, Nadine Kohlhuber, Antje Potthast, Tetyana M. Budnyak, Davide Rigo, Mikhail Balakshin

**Affiliations:** † Department of Bioproducts and Biosystems, School of Chemical Engineering, Aalto University, 02150, Espoo, Finland; ‡ Division of Nanotechnology and Functional Materials, Department of Materials Science and Engineering, Uppsala University, 751 03, Uppsala, Sweden; § Institute of Chemistry of Renewable Resources, Department of Chemistry, University of Natural Resources and Life Sciences (BOKU), 3430, Tulln, Austria

## Abstract

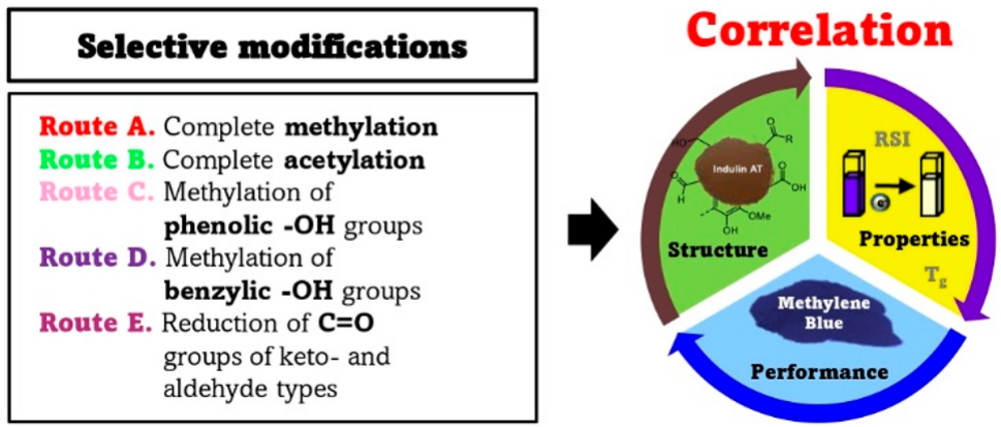

The correlation between lignin structure, its properties, and performance is crucial for lignin engineering in high-value products. Currently, a widespread approach is to compare lignins which differ by more than one parameter (i.e., Kraft vs organosolv vs lignosulfonates) in various applications by attributing the changes in their properties/performance specifically to a certain variable (i.e., phenolic −OH groups). Herein, we suggest a novel approach to overcome this issue by changing only one variable at a time while keeping all others constant before investigating the lignin properties/performance. Indulin AT (Ind-AT), a softwood Kraft lignin, was chosen as the model substrate for this study. Selective (analytical) lignin modifications were used to mask/convert specific functionalities, such as aliphatic (AliphOH) including benzylic −OH (BenzOH) and phenolic −OH (PhOH) groups, carboxyl groups (−COOH) and carbonyl groups (CO) via methylation, acetylation, and reduction. The selectivity and completeness of the reactions were verified by comprehensive NMR analysis (^31^P and 2D HSQC) of the modified preparations together with state-of-the-art molar mass (MM) characterization. Methylene blue (MB) adsorption, antioxidant activity, and glass transition temperature (*T*_g_) were used to demonstrate and compare the properties/performance of the obtained modified lignins. We found that the contribution of different functionalities in the adsorption of MB follows the trend BenzOH > −COOH > AlipOH > PhOH. Noteworthy, benzylic −OH contributes ca. 3 and 2.3 times more than phenolic and aliphatic −OH, respectively. An 11% and 17% increase of *T*_g_ was observed with respect to the unmodified Indulin by methylating benzylic −OH groups and through reduction, respectively, while full acetylation/methylation of aliphatic and phenolic −OH groups resulted in lower *T*_g_. nRSI experiments revealed that phenolic −OH play a crucial role in increasing the antioxidant activity of lignin, while both aliphatic −OH groups and −COOHs possess a detrimental effect, most likely due to H-bonding. Overall, for the first time, we provide here a reliable approach for the engineering of lignin-based products in high value applications by disclosing the role of specific lignin functionalities.

## Introduction

Being one of the most abundant biopolymers on Earth^1^ and a valuable source of aromatic compounds,^2^ lignin is a renewable precursor with a promising forecast in different high value applications, such as thermoplastic polymers, resins, adhesives, sorbents, energy storage, composite materials for tissue engineering and bone regeneration, and carbon fibers/foams.^3−21^ The complex but functionally rich structure of lignin varies according to the original biomass source, the processing conditions during pulping, and the isolation method chosen to separate lignin from the process streams. Technical lignins, such as kraft lignin (KL), result from severe pulping conditions in which reagents like sodium hydroxide and sodium sulfide are involved.^22^ This leads to an altered lignin structure when compared to native lignin.^23,24^ For instance, Kraft pulping involves the hydrolysis of β-O-4 moieties leading to an increase in phenolic hydroxyl groups, and the formation of new C–C linkages within the lignin backbone via condensation reactions.^25,26^ On the other hand, milled wood lignin (MWL), which is considered the benchmark to resemble the structure of native lignin, possesses a higher amount of aliphatic hydroxyl groups, β-5 structures, and a significantly higher amount of β-O-4 interunit linkages.^25^

The lignin behavior relies on different functionalities comprising hydroxyl (−OH), carboxyl (−COOH), and carbonyl (CO), among others. In addition, a broad molar mass distribution (*M*_n_–*M*_*z*_ in the range of 30–130 kDa^27^ and 10–40 kDa^27,28^ for technical and native lignins, respectively) together with the presence of salts/ash and other impurities are other key features to be considered. Such heterogeneity implies that various lignins are expected to behave differently in each specific application.^25,29,30^ Hence, considering such lignin heterogeneity, it cannot be expected that a certain lignin behaves optimally in every application. Therefore, it is critical to define the appropriate niche for each lignin to optimize its performance. This would be a game changer not only from an environmental viewpoint, but also from an economical viewpoint.

The development of a strong approach to lignin engineering is thus of primary importance. It represents a solution to move from petroleum-based products toward green, environmentally friendly, and economically profitable solutions in wood biorefinery with a decreased carbon footprint.^31^ Successful lignin engineering consists of a solid understanding of the correlation among the lignin structure, its properties, and its performance in selected applications. Lignin engineering based on structure–property–performance correlation usually bears numerous samples to be examined, compared, and thoroughly investigated. The current approach is mainly based on the comparison of the performance of chosen lignins from various origins (nonwood, softwood, hardwood) and different processes including traditional pulping methods as well as novel biorefinery concepts in a defined application. In other words, researchers tend to compare the properties of various lignins which differ for more than one variable, making unambiguous conclusions difficult to state.^25,32,33^

In order to overcome this challenge, selective (analytical) modification of lignin should be implemented as the first step in the design of a comprehensive and reliable approach able to provide lignin structure–property–performance correlation.^34−36^ Selective lignin modification aims at changing only one lignin functionality at a time, while keeping all others unmodified (or negligibly modified) prior to a properties/performance evaluation. Currently, such modification methods are still in their infancy. For instance, Sadehgifar et al. performed the selective methylation and oxypropylation of the Kraft lignin,^37^ while others investigated the selective methylation of aliphatic −OH groups of lignin under mild acidic conditions.^34^

The second step in this approach is investigating the effect of each specific modification on the properties or performance of lignin. Noteworthy, to the best of our knowledge, there are no reported examples of the use of selective analytical modification to unequivocally disclose a structure–performance correlation. A similar approach was developed by Gierer and Noren, who investigated the effect of methylation on the rate of the delignification in pulping processes.^38,39^ It provided useful insights on the effect of premethylation (prior to pulping) of softwood shavings and the aryl ether cleavage reactions. In addition, Sadeghifar et al. established the effect of oxypropylation and selective methylation of phenolic hydroxyl groups on thermal properties of the modified lignins which resulted in a decrease in *T*_g_ when the degree of substitution increased.^37^

A powerful method to elucidate the structure of different lignins is a combination of a semiquantitative 2D Heteronuclear Single Quantum Coherence (HSQC) NMR and ^31^P NMR.^1^ The 2D NMR provides information on the structure of lignin with good peak resolution, as well as it allows one to quantify and relatively compare specific functionalities in similar lignin samples. On the other hand, the abundance of hydroxyl groups can be detected and quantified by ^31^P NMR. However, the structural changes in lignin are tightly correlated to its molar mass. The latter affects the lignin performance in terms of its miscibility, solubility, and reactivity. Therefore, the molar mass (MM) and molar mass distributions (MMD) of lignins are essential to establish valid structure–property–performance relationships and are classically obtained by size exclusion chromatography (SEC). The principle of SEC solely relies on the separation of molecules according to their hydrodynamic volume.^40,41^ As a result, a complete description of the MMD is obtained. Conventional SEC requires accurate molar mass calibration, which is particularly difficult for technical lignins due to the lack of appropriate lignin standards.^42−44^ However, the calibration problem can be overcome using molar mass sensitive detectors based on multiangle light scattering (MALS). Therefore, it is of high importance to consider all structural and molar mass changes that occur in lignin to provide a valid correlation between the lignin structure and its performance. For this reason, an effective tool to evaluate the completeness, selectivity and structural alternations is a combination of well-established NMR techniques (2D and ^31^P) and determination of close to absolute molar masses of lignins.

In this context, we propose a reliable approach to address lignin structure–property–performance correlation. Analytical (selective) modification of lignin was performed as the first step in the approach with the aim of masking/modifying one lignin functionality at a time while keeping the structural changes at the minimum. The modifications involved methylation (full, partial, and selective for phenolic −OH), acetylation, and reduction. As a second step, the properties and performance of the modified lignins were evaluated. The effect of each functionality on lignin sorption capacity, *T*_g_ and the antioxidant activity was established and discussed. For the first time, clear trends have been unequivocally disclosed.

## Experimental Section

### Materials and Chemicals

Dimethyl sulfate (DMS), sodium hydroxide (NaOH), sulfuric acid (95.0–98.0%), anhydrous methanol (99.8%), anhydrous dioxane (99.8%), *p*-toluene sulfonic acid, sodium borohydride (NaBH_4_), ethanol (99.9%), acetone (≥99.5%), hydrochloric acid (HCl), acetic anhydride, deuterated chloroform (CDCl_3_), pyridine, deuterated dimethyl sulfoxide (DMSO-*d*_6_), endo-*n*-hydroxy-5-norbornene-2,3-dicarboximide (e-HNDI), chromium(III) acetylacetonate (Cr(acac)_3_), and 2-chloro-4,4,5,5-tetramethyl-1,3,2-dioxaphospholane (TMDP; all analytical grades) were purchased from Sigma-Aldrich. 1,1-Diphenyl-2-picrylhydrazyl stable radical was purchased from Thermo Fisher. Softwood Kraft lignin (Indulin AT) is a commercially available technical lignin. Prior to conducting the experiments, Indulin AT (Ind-AT) was dried under a vacuum overnight with the aid of P_2_O_5_. Modifications of the lignin were carried out using reported protocols.^34,37^

### Complete Methylation of All −OH/–COOH Groups (Ind-DMS)

Complete methylation of lignin was performed using a procedure proposed by Zakis.^34^ A total of 1 g of Ind-AT was dissolved in 10 mL of 1 M NaOH. When dissolved, solid NaOH pellets were added to form a 30 wt % NaOH solution. Then, 15 mL of freshly prepared 30 wt % NaOH was added. Subsequently, 10 mL of DMS was added dropwise within 30 min and the mixture was kept under continuous stirring for 24h. The mixture was diluted 3-times with deionized water (100 mL). The modified lignin was precipitated by adding a 1 M solution of sulfuric acid until pH 2 and isolated by filtration using a glass crucible (pore size 10–16 μm). Completely methylated lignin (Ind-DMS) was exhaustively washed with deionized water (200 mL) until neutral pH and was exposed to air drying followed by vacuum oven drying (*T* = 40 °C, *p* = 0.1 mbar) in the presence of P_2_O_5_. The procedure was repeated 3 times to maximize the degree of methylation of all −OH groups. Ind-DMS was obtained in 78 wt % yield with respect to the initial material and characterized by ^31^P NMR and SEC-MALS techniques.

### Complete Masking all OH Groups by Acetylation (Ind-Ac)

Lignin acetylation was performed according to the procedure reported elsewhere.^34^ A total of 1 g of Ind-AT was dissolved in 5 mL of pyridine and stirred until fully dissolved. After that, 5.9 mL of acetic anhydride was added, and the resulting mixture was stirred for 48 h at room temperature. The mixture was then diluted with 30 mL of EtOH and the solvent was rotary-evaporated. The acetylated lignin (Ind-Ac) underwent the conventional drying procedure described above. The yield of the acetylated sample was 99% and was characterized by 2D HSCQ NMR and SEC-MALS techniques.

### Selective Methylation of Phenolic −OH Groups (Ind-Ph)

DMS was used to selectively methylate all PhOH groups in lignin under controlled pH as proposed by Sadehgifar et al.^37^ Briefly, 1 g of Ind-AT was dissolved in 15 mL of 0.7 M NaOH. DMS (1 mL, 2.5:1 = molDMS:molPhOH) was added to the mixture and then stirred for 30 min at room temperature. Subsequently, the solution was heated for 2 h at 80 °C. In order to avoid unwanted precipitation, the pH value of the reaction was kept in the range from 11.0 to 11.5 by continuous addition of the 0.7 M NaOH solution. Lignin was precipitated by acidifying the mixture with 1 M HCl until pH = 2, filtered on a glass crucible (pore size 10–16 μm), exhaustively washed with deionized water (200 mL) and dried as described above. The selectively methylated lignin (Ind-Ph) was isolated in 90 wt % yield based on the initial material and characterized by ^31^P NMR and SEC-MALS techniques.

### Methylation of Benzylic −OH Groups and Esterification (Ind-Me)

The aim of this experiment was to selectively methylate the benzylic hydroxyl groups of Ind-AT following the procedure described elsewhere.^34^ A total of 1 g of Ind-AT was dissolved in 14.4 mL of anhydrous dioxane (99.8% purity). A total of 14.4 mL of 0.3 M *p*-toluene sulfonic acid solution in methanol was added dropwise under vigorous stirring until homogeneity. The mixture was kept at room temperature for 4 days, and then the modified lignin was precipitated by diluting the lignin solution with deionized water (288 mL) dropwise under vigorous stirring, until the water:dioxane ratio reached 20:1. Modified lignin was obtained by filtration on the glass crucible (pore size 10–16 μm) and exhaustively washed with deionized water until neutral pH was reached (200 mL). α-Benzylic methylated lignin (Ind-Me) was first air-dried, followed by vacuum oven drying (*T* = 40 °C, *p* = 0.1 mbar) in the presence of P_2_O_5_. It was obtained in 90 wt % yield and characterized by 2D HSQC NMR and SEC-MALS techniques.

### Reduction of Carbonyl Groups of Keto and Aldehyde Type (Ind-R)

The aim of the current experiment is the complete reduction of carbonyl functional groups of lignin, and it was performed according to the method proposed by Zakis.^34^ A total of 1 g of Ind-AT was dissolved a mixture of 0.1 M NaOH (20 mL) + EtOH (40 mL). Following that, 60 mL of 0.8 wt % NaBH_4_ (aq) solution was added, and the resulting mixture was stirred for 16 h at room temperature. Additional 0.2 g of NaBH_4_ (powder) was added to the reaction mixture and stirred for 16 h at room temperature. Then, the reduced lignin was precipitated by adding 1 M HCl solution dropwise under vigorous stirring until the pH 2. Subsequently, lignin suspension was frozen, thawed, and filtered on a glass crucible (pore size 10–16 μm). A freezing–thawing procedure facilitated the lignin filtration due to physical cross-linking of the lignin macromolecules.^45,46^ The solid precipitate was exhaustively washed with deionized water (200 mL) and then dried, as described above. Reduced lignin (Ind-R) was isolated in 66 wt % yield based on the initial material and characterized by 2D HSQC NMR and SEC-MALS techniques.

### NMR Analysis

#### ^31^P NMR

The quantification of different hydroxyl groups in the modified lignins was performed by ^31^P NMR spectroscopy according to the method recently optimized by our group.^47^ The measurements were executed on a Bruker NMR Spectrometer AV III 400 with an acquisition time of 1 s and the relaxation delay of 5 s, while the number of scans was set to 128. To 40 mg of the lignin sample, 0.4 mL of a freshly prepared mixture of pyridine and CDCl_3_ (1.6:1, v/v) was added. After that, 100 μL of e-HNDI solution (0.3 μmol mg^–1^) used as an internal standard (IS) and 50 μL of chromium(III) acetylacetonate solution (11.4 mg mL^–1^) were added to the vial with the lignin solution. In addition, 100 μL of derivatization agent (TMDP) was added to the mixture. The vial was vortexed until the mixture became homogeneous, and the latter was transferred into the NMR tube for the NMR acquisition. The obtained spectra were phased and calibrated based on the signal of the 2,2′-oxybis(4,4,5,5-tetramethyl-1,3,2-dioxaphospholane) water-derivatized product at 132.2 ppm. A linear function was implemented to correct for a baseline.

Determination of hydroxyl groups in reduced lignin was performed following the procedure proposed by Stücker et al.^48^ Briefly, approximately 20 mg of the sample was dissolved in 600 μL of DMF:pyridine = 2:1 solution. 100 μL of e-HNDI solution in DMF (50 mg mL^–1^) and chromium(III) acetylacetonate solution (5 mg mL^–1^) was added to the reduced lignin solution. Following that, 100 μL of the phosphorylating agent (TMDP) and 200 μL of CDCl_3_ were added. The acquisition procedure and the spectrum processing were carried out as described above.

#### 2D HSQC NMR

The 2D NMR spectra of the selected modified lignins were obtained using a Bruker AVANCE 600 NMR spectrometer equipped with a CryoProbe. Approximately 75 mg of the sample was dissolved by adding 0.6 mL of DMSO-*d*_6_. ^1^H-dimension parameters were defined by an acquisition time of 77.8 ms and the number of scans equal to 36 per block. Data was collected using the 1024 collected complex points. The ^13^C dimension was characterized by the acquisition time set to 3.94 ms, and 256-time increments were recorded. The 2D HSQC NMR data was processed by 1024 × 1024 data points along with the Qsine function, which was employed for both ^1^H and ^13^C dimensions. The calibration of the chemical shifts was performed based on the DMSO peak cross-signal at δ_C_/δ_H_ 39.5/2.49 ppm/ppm. The cross-peaks reflected on the spectra were assigned based on the previous reports.^22,35^ Different functionalities were quantified by assuming that the sum of G- and S-units is 100 and can be expressed as follows:
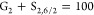


The amount of each functionality is defined in mol % per 100 aromatic units (Ar).

#### Determination of MWD with SEC/MALLS-785/RI in DMSO/LiBr

The molar mass analysis was done by means of multiangle light scattering (MALS) in accordance with Zinovyev et al.^42^ In brief, 10 mg of lignin was dissolved in 1 mL of DMSO/LiBr (0.5% w/v). After complete dissolution, the sample was filtered through a 0.45 μm PTFE syringe filter. The SEC analysis was performed on an Ultimate 3000 system, consisting of autosampler column oven (all Thermo Fisher Scientific Inc., Waltham, MA, U.S.A.), HPLC Pump Series P580 (Dionex Softron GmbH, Germering, Germany), HELEOS I MALS detector operating at 785 nm, and an Optilab T-rEX differential refractive index detector, λ = 633 nm (all Wyatt Technology, Santa Barbara, U.S.A.) under the following conditions: 35 °C column temperature, 10 μL injection volume, 0.5 mL min^–1^ flow rate, and 65 min run time. For the separation, three Agilent PolarGel M columns (7.5 × 300 mm with 5 μm particle size) and a precolumn (7.5 × 50 mm) were used. The data was processed with Astra 7.3.

#### Methylene Blue Adsorption

To evaluate the performance of the modified lignins, a set of sorption experiments was performed. The experiment was carried out according to Budnyak et al.^14^ To specify, a precisely weighted adsorbent sample (∼0.05 g) of lignin sample was placed into the flask containing a methylene blue (MB) solution with a known initial concentration. The flask was then shaken in an Orbital Shaker INC/REFRIG 5000IR (*rcf* = 0.24 × g) and 25 °C. Once adsorption occurred, the liquid and solid phases were separated via centrifugation at 3374 × g for 5 min on a Heraeus Megafuge 40 Centrifuge (ThermoScientific). The residual dye concentration in the equilibrium aqueous phases was determined using spectrophotometry on a UV-3100PC spectrophotometer with a square cuvette (optical path length *l* = 1 cm at λ = 664 nm). The removal efficiency (*R*, %) and specific concentration of the adsorbed MB (*q*_*e*_, mol g^–1^) were calculated as
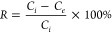
1
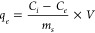
2where *C*_*i*_ and *C*_*e*_ are the initial and equilibrium MB concentrations, *m*_*s*_ (g) is the weight of lignin sample, and *V* (L) is the volume of the initial dye solution. Each test was replicated three times.

#### Differential Scanning Calorimetry

Differential scanning calorimetry (DSC) was employed to measure the glass transition temperature (*T*_g_) of the lignin samples. The thermograms were recorded on a Discovery DSC 250 differential scanning calorimeter (TA Instruments, U.S.A.). Each sample of approximately 7 mg was heated under a nitrogen flow of 50 mL min^–1^ at the heating rate of 10 °C min^–1^ within a temperature range from 25 to 200 °C and held for 5 min. After that, the sample was cooled to 25 °C at the same rate. Finally, the sample was heated from 25 to 220 °C at a heating rate of 10 °C min^–1^. The *T*_g_ of each sample was determined based on the second heating curve using TA Universal Analysis software. The DSC curves are reported in the SI section.

#### Radical Scavenging Activity

The antioxidant activity of each modified lignin was evaluated using 2,2-diphenyl-1-picrylhydrazyl (DPPH) as a reactive free radical by adjusting previously reported procedures.^38,39^ All the solutions were subjected to UV–vis spectroscopy on a Shimadzu UV-2550 spectrophotometer by placing a solution into a 10 mm length quartz cuvette. Each lignin sample was dissolved in a 90 vol % acetone (aq) to form a set of solutions with concentrations in the range 120–600 mg L^–1^. Each solution was mixed with a 75 μmol L^–1^ solution of DPPH in 90 vol % acetone (aq) in a lignin/DPPH = 1:39 ratio. The absorbance of the prepared solutions was analyzed at 515 nm wavelength at 0 time point (immediately after the solution preparation) and after 24 h, when steady state was reached. In addition, the absorbance over time of a blank solution (75 μmol L^–1^ solution of DPPH in 90 vol % acetone (aq)) was measured to evaluate the self-degradation of DPPH at 0 time point and after 24 h. More details on the experimental procedure are reported in SI.

## Results and Discussion

### General

**Figure 1:**
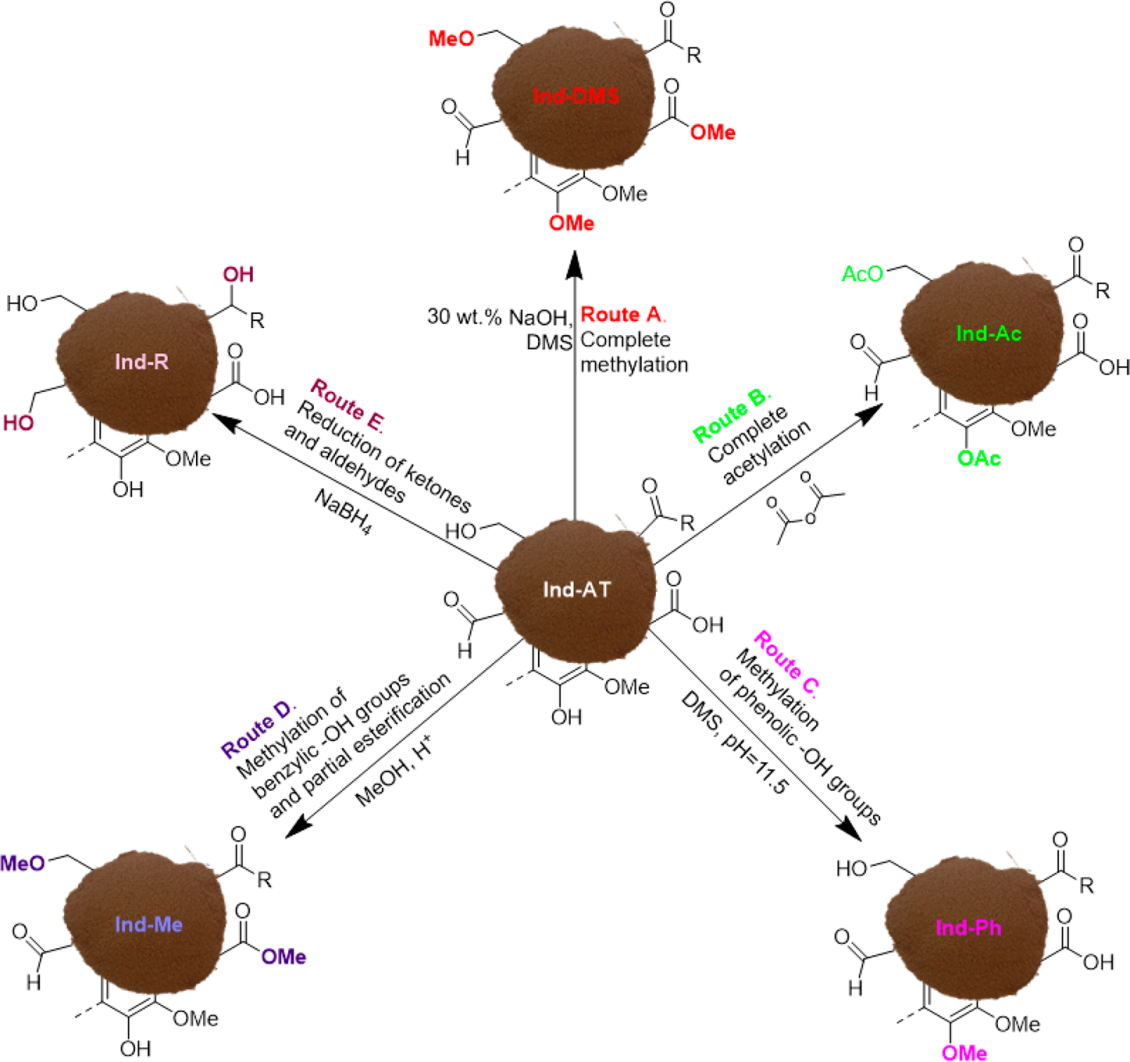
Routes for the selective modifications of Ind-AT. Route A: Complete methylation of all −OH/–COOH groups; Route B: Complete masking all −OH groups by acetylation; Route C: Selective methylation of PhOH groups; Route D: Methylation of benzylic −OH groups and esterification; Route E: Reduction of carbonyl groups of keto and aldehyde type.

The aim of the current work is to establish the structure–property–performance correlation by selectively modifying/masking one specific lignin functionality at a time followed by the evaluation of the effect of each modification on (i) lignin performance as a sorbent for methylene blue (MB) and (ii) on its properties, such as glass transition temperature (*T*_g_) and radical scavenging activity (RSI). Indeed, the production of sorption-active materials represents a valuable field of application for lignin,^40^ while the thermal behavior and the antioxidant activity are important properties for the production of (co)polymers, blends and composites using different formulations, respectively.^41,42^ In addition, they are fast screening options to demonstrate the importance of certain lignin functionalities. Indulin Kraft lignin (Ind-AT) was used as a model substrate for this work. Ind-AT was exposed to a set of diverse chemical modifications (Figure 1): (a) complete methylation (Ind-DMS); (b) complete acetylation (Ind-Ac); (c) methylation of phenolic −OH (PhOH) groups (Ind-Ph); (d) methylation of benzylic −OH (benzOH) groups (Ind-Me); (e) reduction of C=O groups of keto and aldehyde types (Ind-R).

### Characterization of the Modified Lignins by ^31^P and 2D HSQC NMR

#### Complete Methylation of All −OH/–COOH Groups (Ind-DMS)

**Figure 2:**
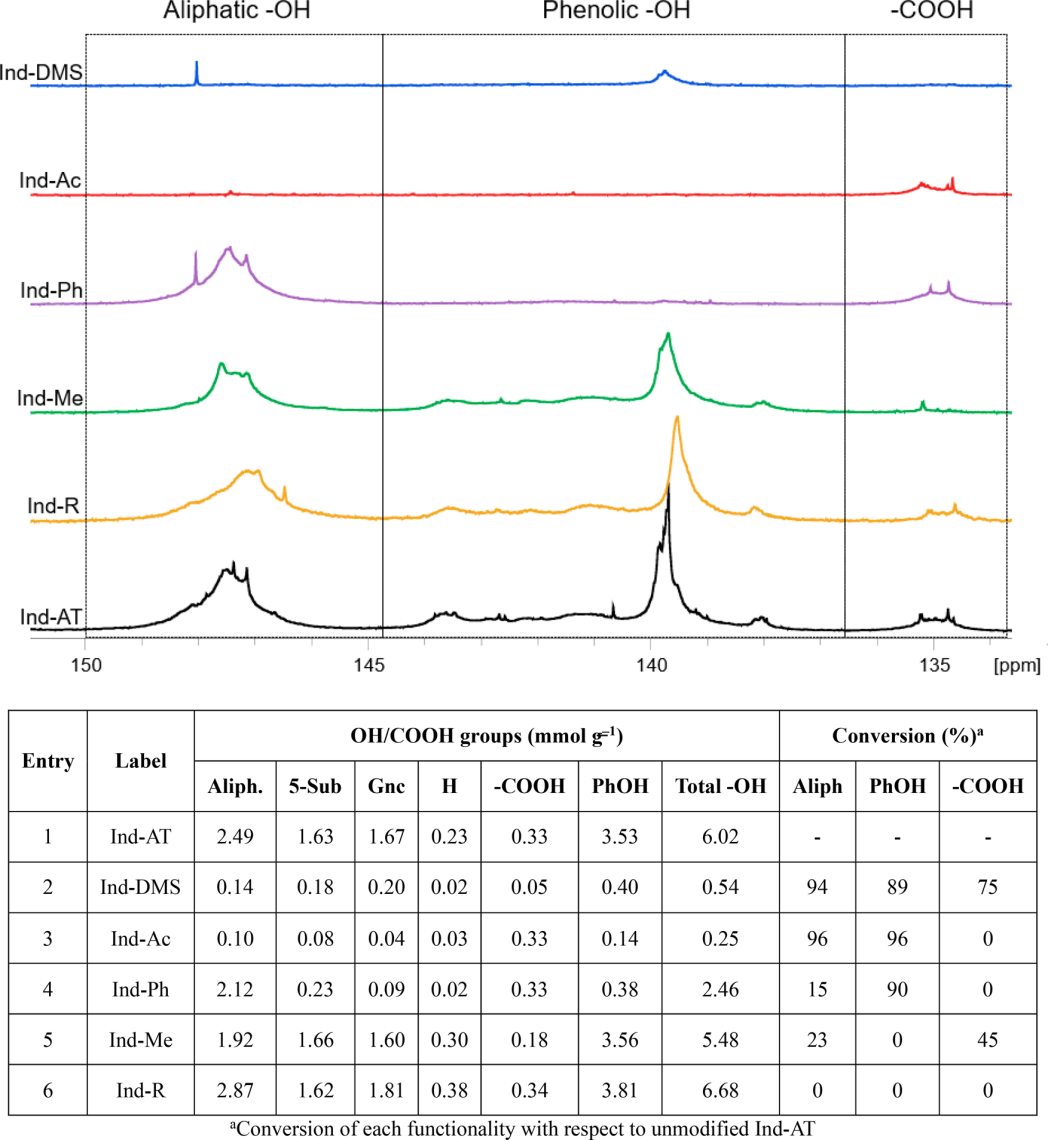
Top: ^31^P NMR spectra of the modified lignins and reference lignin (Ind-AT). Bottom: quantification of −OH groups in the modified lignins by ^31^P NMR.

With the aim of masking all the −OH/–COOH functional groups of Ind-AT, methylation with dimethyl sulfate (DMS) was performed by adjusting the protocol reported by Zakis et al.^34^ The procedure was repeated three times to maximize the degree of conversion of −OH/–COOH groups of Ind-AT. Data obtained from ^31^P NMR analysis resulted in the etherification of aliphatic and phenolic −OH groups with 94% and 89% conversion, respectively (Figure 2). In addition, −COOH groups were converted as well (85%), most likely into their corresponding methyl esters. Overall, the three subsequent methylation steps allowed for good conversion of −OH (both aliphatic and phenolic), while only 0.05 mmol g^–1^ of residual −COOH is present in Ind-DMS after methylation.

#### Complete Masking All OH Groups by Acetylation (Ind-Ac)

Lignin acetylation allowed for masking of all hydroxyl groups (PhOH+AliphOH) present in lignin without modifying −COOH groups. Acetylation mixture consisting of equimolar amounts of Ac_2_O and pyridine was chosen as the most appropriate for lignin acetylation.^34^ The completeness and selectivity of the reaction were proven by ^31^P NMR. The analysis showed the absence of aliphatic −OH and phenolic −OH groups (Figure 2; top). The conversion calculated for both aliphatic and phenolic −OH was 96%, while −COOH was not converted (Figure 2; bottom, entry 3).

#### Selective Methylation of Phenolic OH Groups (Ind-Ph)

Phenolic −OH groups were methylated using DMS as the methylating agent. Based on previously reported procedures,^37^ the pH of the reacting mixture should be kept in the range of 11.0–11.5 and the temperature below 80 °C. Hence, higher pH and *T* values promote hydrolysis of DMS. In turn, H_2_SO_4_ is produced as a byproduct, causing a sharp drop in the pH of the reaction medium which promotes lignin precipitation. To avoid this, Sadehgifar et al. suggested to control the pH by constantly adding 0.7 M NaOH solution to the mixture.^37^ It allows for balancing between unwanted ionization of aliphatic −OH groups and the acceptable pH of the solution needed for selective methylation of PhOH yet avoiding lignin precipitation while methylating. The presence of a very weak and broad array of signals in the range 145–136.8 ppm in the ^31^P NMR spectrum (Figure 2) stands for successful methylation of PhOH groups of Ind-AT, with a residual PhOH groups content of 0.38 mmol g^–1^, which represent 10% of the initial amount (Figure 2; bottom, entry 4). Parallel to this, 15% conversion of aliphatic −OH was detected as well. Overall, the conversion of PhOH was ca. 6 times higher with respect to aliphatic −OH, which means that a selectivity of 84% was achieved.

#### Methylation of Benzylic OH Groups and Esterification (Ind-Me)

**Figure 3:**
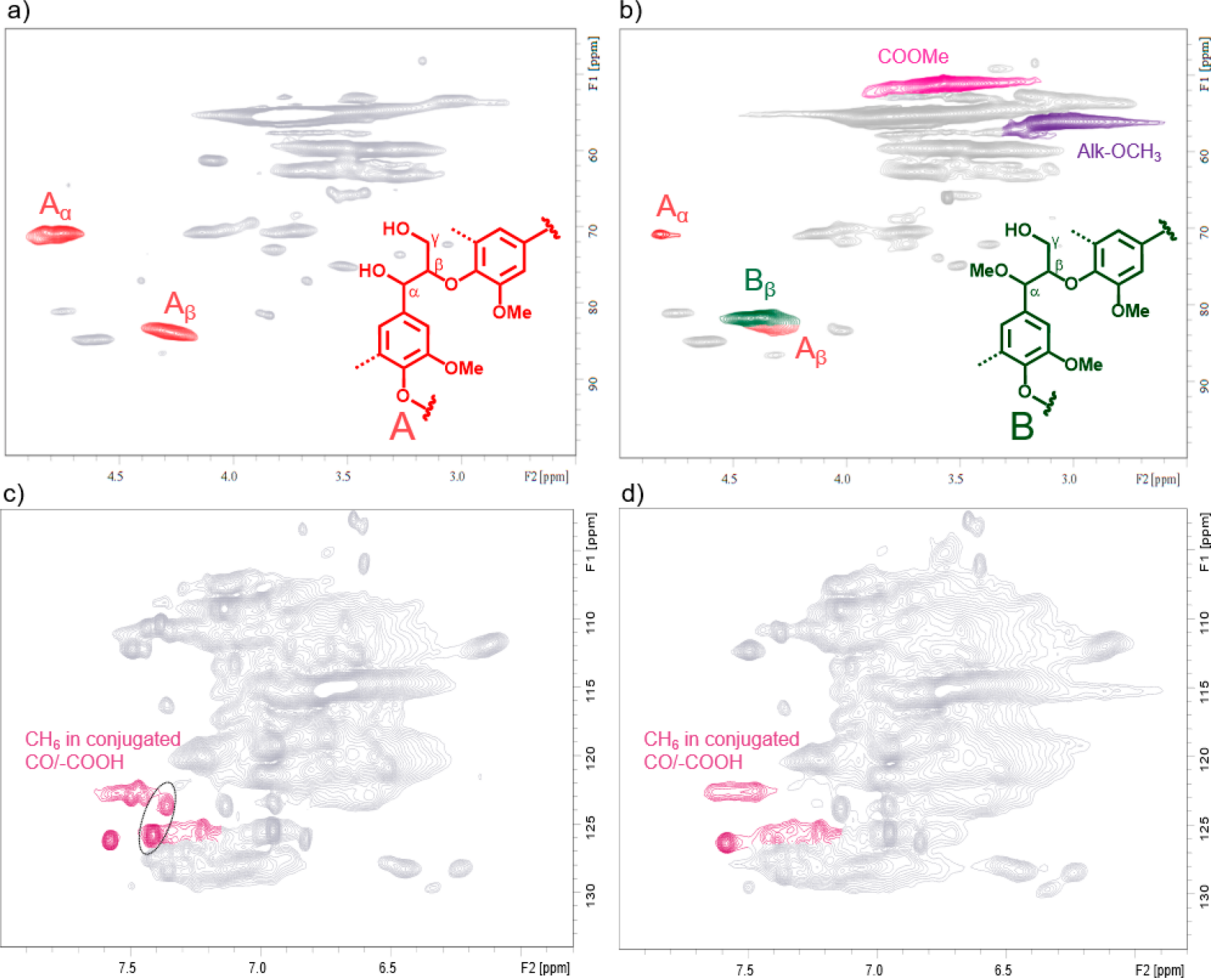
HSQC NMR spectrum of the modified lignins: (a, c) Ind-AT, (b) Ind-Me, and (d) Ind-R.

To selectively methylate benzylic −OH (BenzOH) groups, the reaction was carried using methanol (MeOH) as methylating agent and *p*-toluene sulfonic acid as an acidic catalyst at room temperature.^34^ The outcome of the reaction was evaluated by 2D HSQC NMR (Figure 3a,b and Table 1). The assignment of signals in the HSQC spectra discussed below is based on the previously reported works.^22,35^ The spectrum shows new cross-peaks corresponding to R-OCH_3_ groups at δ_C_/δ_H_ 58.0–55.7/3.2–2.5. Parallel to this, a new signal appeared at δ_C_/δ_H_ 83.84/4.27 ppm, which is assigned to β-CH in β-O-4/α-OMe structures (structure B). A detailed quantification revealed that the intensity of β-O-4/α-OH signals dropped significantly from the reaction, which resulted in the 78% conversion of β-O-4/α-OH achieved after 4 days, which was consistent with the slight decrease of aliphatic −OH groups observed in the ^31^P NMR analysis (Figure 2). This is consistent with a conversion of β-O-4/α-OH structures into the corresponding methyl ethers (β-O-4/α-OMe). The latter result did not well correlate with the decrease of β-CH in β-O-4/α-OH signal (53% conversion), most probably due to partial overlap with the signal of β-CH in β-O-4/α-OMe and other benzyl ether-type structures. Additionally, a new intense signal appeared at δ_C_/δ_H_ 52.1–49.9/3.9–3.3 ppm, which was assigned to methyl esters of different types (RCOOMe). The conversion of −COOH groups was evaluated by ^31^P NMR analysis (45%; Figure 2).

**Table 1:** Quantification of Key Ind-Me Moieties by 2D HSQC NMR (per 100 Ar)

assignment	chemical shift, ppm (δ_C_/δ_H_)	Ind-AT	Ind-Me	conversion^a^ (%)
CHα in β-O-4/α-OH	73.0–73.9/5.0–4.6	9.5	2.2	78
CHβ in β-O-4/α-OH	85.9–81.3/4.5–4.1	7.3	3.4	53
Alk-OCH_3_	58.0–55.83.2–2.5	0	11.3	
CHβ in β-O-4/α-OMe	82.5–80.5/4.6–4.3	0	10.8	
R-COOMe	52.1–49.9/3.9–3.3	0.8	9.3	

a Conversion of each functionality with respect to unmodified Ind-AT.

#### Reduction of Carbonyl Groups of Keto and Aldehyde Type (Ind-R)

Balakshin et al. found that reduction in acidic conditions resulted in a quick decomposition of NaBH_4_ and promoted the occurrence of side reactions, such as the degradation of vinyl ether moieties in the soda lignin.^35^ In light of this, we here performed the reduction of Ind-AT in weak alkali medium, by adapting our previously reported procedure (see also experimental).^34^ With the aim of increasing the number of −OH groups in the Ind-R sample, ketos and aldehydes of different types were the targeted groups. The quantification of the Ind-R −OH/–COOH functionalities was performed by ^31^P NMR (Figure 2), and the sample was further analyzed by 2D HSQC (Figure 3 c and d). A 15% increase in the number of aliphatic −OH groups from 2.49 mmol g^–1^ to 2.87 mmol g^–1^ was observed by ^31^P NMR analysis (Figure 2; bottom, entry 6). The number of −COOHs was not affected by the reductive treatment, while an 8% increase was observed for phenolic −OH (Figure 2). Such small increase in phenolic −OH groups can be attributed to the loss of low molar mass fractions during lignin isolation via precipitation, as discussed in the following section. According to Balakshin et al., signals of β-O-4 structures with α-CO groups were not found in lignin preparations after pulping and, consequently, such structures cannot be unambiguously assigned and might be located in terminal side chains.^22^ Nevertheless, the conversion of α-CO groups through reduction can be indirectly observed by the increase in α-OH moieties assigned by the CHα signal in β-O-4/α–OH structures at δ_C_/δ_H_ 73.0–68.6/5.1–4.6 ppm from 9.5 to 9.9/100 Ar (Table 2). In addition, another area of interest in the HSQC spectrum for the detection of conjugated CO/–COOH moieties is located in the range of δ_C_/δ_H_ 122–126/7–7.6 ppm (Figure 3c,d).^22^ Even though an unambiguous peak assignment in this region is complicated due to the inability to accurately distinguish between carbonyl signals and carboxyl ones, the combination of ^31^P NMR and HSQC data allows us to indirectly draw certain conclusions. Hence, since a 32% decrease in the amount of conjugated α-CO/-COOH moieties from 7.1 to 4.8/100 Ar was detected (Table 2) while the amount of −COOHs remained unchanged (Figure 2), this means that a reduction of CO groups of keto and aldehyde types occurred.

**Table 2:** Quantification of Key Ind-R Moieties by HSQC NMR (per 100 Ar)

assignment	chemical shift, ppm (δ_C_/δ_H_)	Ind-AT	Ind-R	conversion^a^ (%)
β-O-4/α-OH	73.0–68.6/5.1–4.6	9.5	9.9	
conjugated α-CO/-COOH (total)	126.6–122.0/7.6–7.0	7.1	4.8	32

a Conversion of each functionality with respect to unmodified Ind-AT.

#### Molar Mass Distribution (MMD)

**Figure 4:**
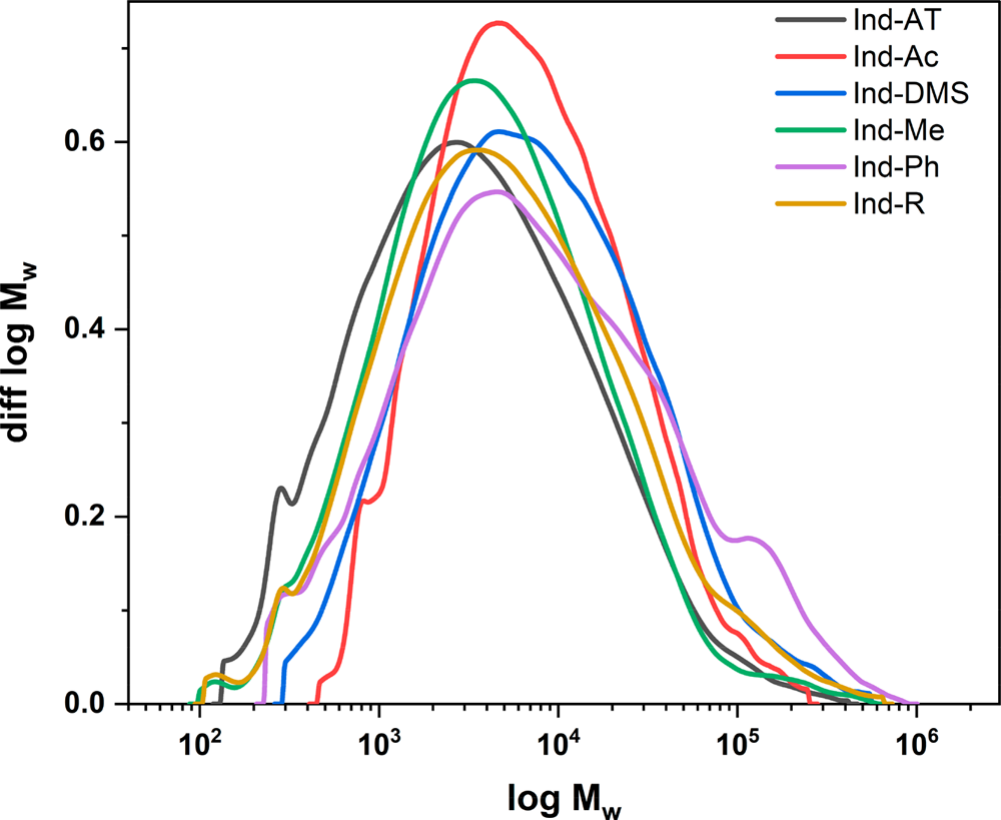
Molar mass distributions of the modified samples and Ind-AT.

The molar mass (MM) has a significant influence on the physiochemical properties of technical lignins and is crucial to establish valid structure–property–performance correlations.^41,49^ Consequently, we analyzed the initial substrate (Ind-AT) and modified lignins by SEC-MALS_785nm_-RI to understand the influence of distinct modifications (i.e., acetylation, methylation, and reduction) on the MM. Due to the modifications selectivity and mild reaction conditions, only a slight increase in MM, caused by the introduction of methoxy or acetyl groups, is expected. However, when comparing the molar mass distributions (MMDs; Figure 4) before and after modification, a significant increase in statistical moments of the MMD (Table 3) is observed, independent of the type of modification. This rise in MM is caused either by the reaction or by the loss of material during sample isolation. Therefore, we repeated exemplarily the reduction procedure without the reducing agent to see if the change in the MM was caused by the purification (e.g., precipitation and filtration) after the reaction. As a result, we could observe an increase in MM that corresponds to the rise of *M*_n_ after methylation and reduction (Table S1 and Figure S1). Consequently, we propose that the increase in the observed MM is mainly caused by the loss of low MM fractions during precipitation and filtration, which should be considered when discussing the results. However, in the case of acetylation, a different isolation procedure (99% yield) was used. Therefore, we propose that rise in *M*_n_ after acetylation is mainly caused by introducing acetyl groups due to the better accessibility and higher abundance of −OH groups in the low MM region. Furthermore, we see that the complete methylation (Ind-DMS) and the methylation of PhOH groups (Ind-Ph) cause aggregation and, concurrently, a higher *M*_*z*_ (Figure 4).

**Table 3:** Samples Overview and Calculated Statistical Moments

sample	*M*_n_ (Da)	*M*_w_ (Da)	*M*_*z*_ (Da)	*Đ*
Ind-AT	1400	10100	64300	7.28
Ind-Ac	3700	14200	51300	3.79
Ind-DMS	3100	19600	106700	6.33
Ind-Me	1800	11500	89700	6.42
Ind-Ph	2500	29000	171500	11.60
Ind-R	1900	16800	121200	8.92

Overall, the advanced characterization of the lignin samples discussed in the previous sections demonstrated that the analytical modifications occurred selectively with neglectable effects on their molar mass distribution which were mainly due to workup stages and not to the modification of the lignin structure. In addition, TGA analysis proved that the ash content in all the lignin samples is below 10% (Figures S2 and S3). In light of this, we propose that its influence is negligible compared to the effect of the chemical modification.

### Structure–Performance Correlation

#### Effect of the Modifications on the MB Adsorption

**Figure 5:**
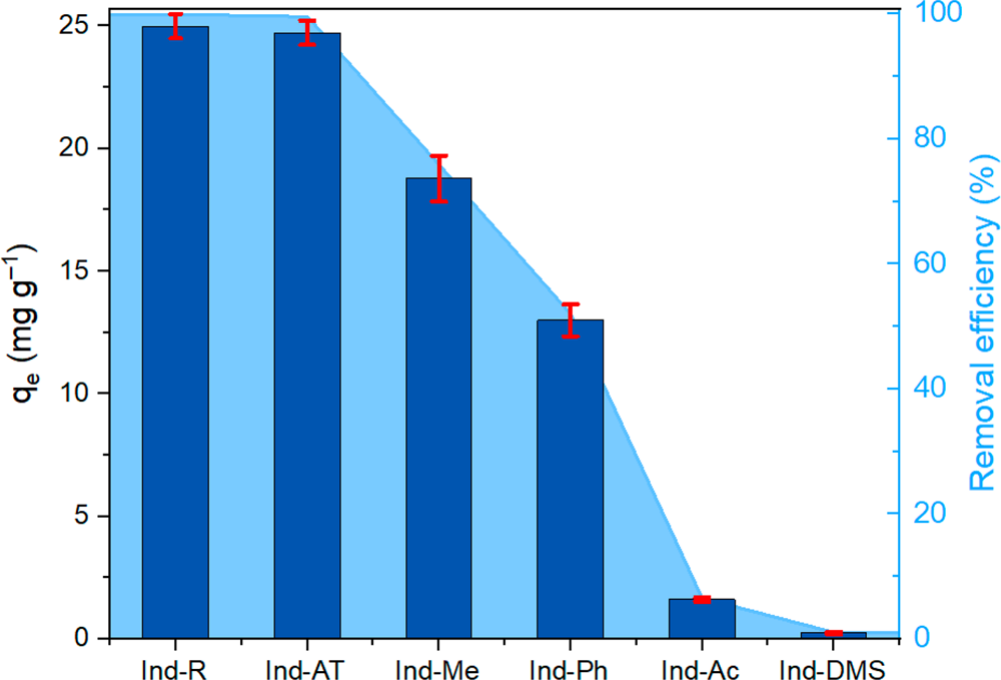
Comparison of the adsorption behavior of bare and modified Ind-AT in the solution containing 44.4 mg L^–1^ of MB.

This study aimed to investigate the contribution of different functional groups in the lignin sorption capacity. To achieve this goal, the performance in MB dye removal of the modified lignin samples was compared with the control sample (Ind-AT; Figure 5).

**Figure 6:**
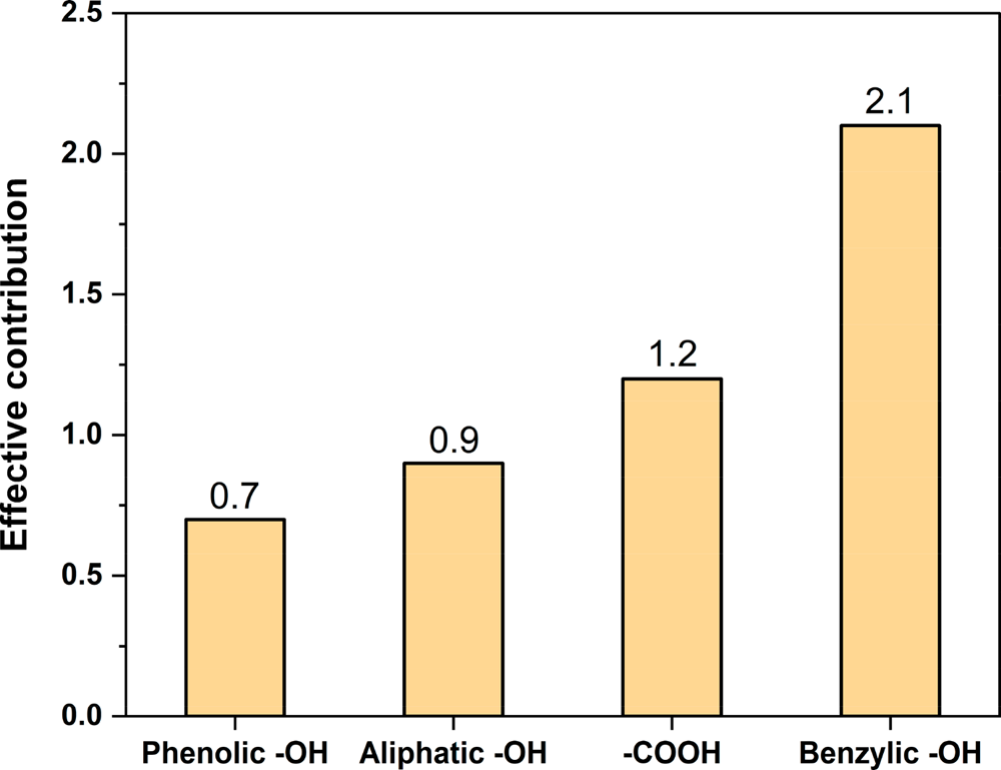
Effective contribution (EC) of the lignin functionalities in MB adsorption.

The results showed that the contribution of −OH groups (both aliphatic and phenolic) to the lignin sorption capacity was crucial. Complete elimination of all −OH groups through methylation and acetylation led to a decrease of more than 97% in the adsorption activity at equilibrium (*q*_*e*_) of the samples, indicating that −OH groups played a dominant role in the sorption process. This is additionally confirmed by the fact that the reduction of carbonyl groups to −OH groups (Ind-R sample) resulted in the highest removal efficiency of 99.9% compared to the reference Ind-AT and all other modified samples. To better compare and discuss the effect of each specific functionality, the effective contribution (EC_*i*_), normalized contribution, of each moiety should be calculated considering their amount in Ind-AT. So, the EC_*i*_ was calculated as follows:
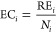
3where RE_*i*_ is the removal efficiency of each lignin functionality and *N*_*i*_ is an amount of the functionality in the reference (Ind-AT) sample determined either by ^31^P NMR or by HSQC (Figure 2 and Tables 1 and 2) expressed per each 100 Ar. Figure 6 summarizes the EC_*i*_ values for key lignin functionalities.

The removal efficiency of phenolic −OH (RE_PhOH_) and, thus, the EC_PhOH_, can be straightforwardly calculated according to eq 4, since phenolic −OH are the only masked groups in the Ind-Ph sample (Figure 2):
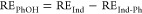
4where RE_Ind_ is the removal efficiency of Ind-AT set as a reference to 100 and RE_Ind-Ph_ is the removal efficiency obtained for the Ind-Ph sample (Figure 5). Based on eqs 3 and 4, EC_PhOH_ = 0.7.

To calculate the EC_*i*_ for other functionalities, some assumptions are needed. Hence, the removal efficiency of aliphatic −OH (RE_AlipOH_) can be indirectly calculated as follows:
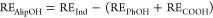
5

To calculate the RE_COOH_:
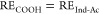
6

The latter assumption can be stated as the −COOH groups are the sole residual functionalities in the Ind-Ac sample (Figure 2). So, based on eqs 3–6, EC_COOH_ and EC_AlipOH_ are 1.2 and 0.9, respectively. Then, other assumptions must be made to calculate the effective contribution for benzylic −OH in β-O-4/α-OH structures (EC_BenzylOH_). Thus, the removal efficiency of benzylic −OH can be expressed as
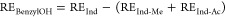
7

This is true since in the Ind-Me sample the masked groups are β-O-4/α-OH structures through methylation and −COOH via esterification (Table 1 and Figure 3), while RE_COOH_ = RE_Ind-Ac_, according to eq 6. So, based on eq 7, EC_BenzylOH_ = 2.1.

Overall, the EC_*i*_ values suggest that the activity of each functionality in the adsorption of MB follows the trend benzylic −OH > −COOH > AlipOH > PhOH (Figure 6). Intriguingly, the EC of benzylic −OH is 3 times higher than the one of phenolic −OH. This interaction could occur through electrostatic interaction, π–π interactions, or H-bonding; however, this question is worth to be studied in detail in a separate study. Overall, the latter findings provide insights into the critical role of different functional groups in lignin sorption capacity and can contribute to the development of more efficient sorbents.

As a conclusive discussion, it should be mentioned that semiquantitative HSQC experiments were used to evaluate the structural changes occurring after acidic methylation (Figure 1, Route D) and reduction (Figure 1, Route E). Nevertheless, even though it is well-known that HSQC is not quantitative at an absolute level due to different relaxation times of different functionalities,^50,51^ it properly works to relatively compare the results of similar lignin units in similar lignin samples, as in the present work. In addition, we recently found good correlation between HSQC and quantitative ^13^C NMR results in the quantification of certain lignin functionalities.^52,53^

### Structure–Property Correlation

#### Effect of the Modifications on the RSI

**Scheme 1:**
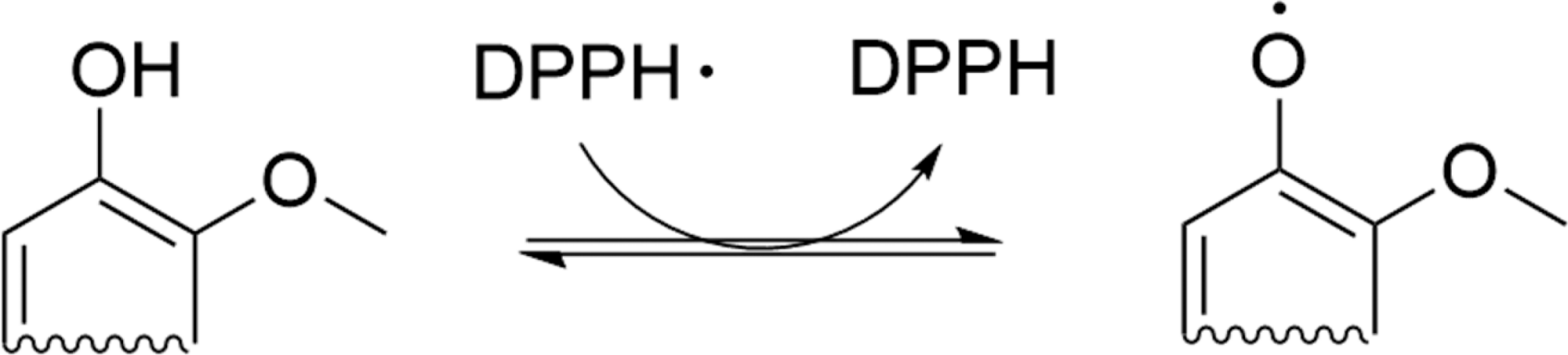
Reported Mechanism for the DPPH Radical Scavenging

**Figure 7:**
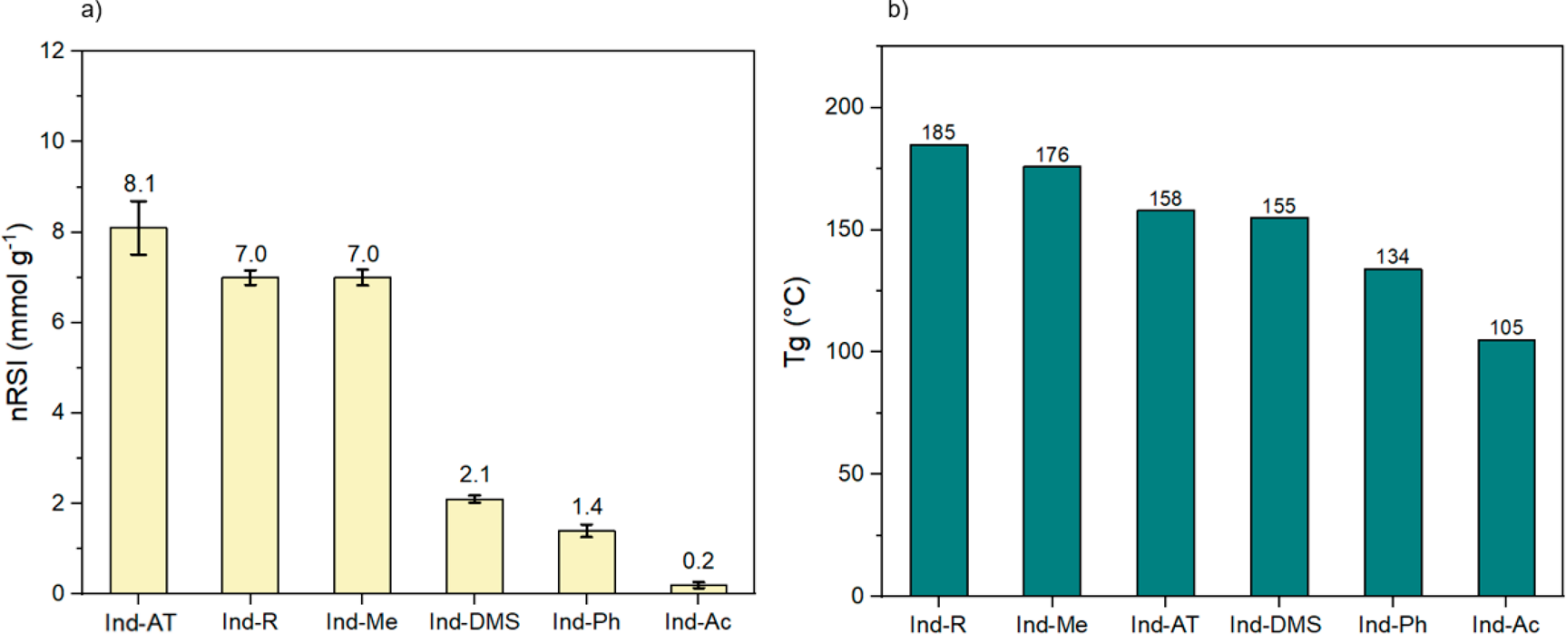
Effect of the selective modifications on lignin properties: (a) nRSI values of the modified lignins and the reference Ind-AT: radical scavenging activity decreases with an increase in the amount of masked −OH/–COOH groups; (b) *T*_g_ values of the modified lignins and Ind-AT: increase in the amount of etherified PhOH moieties resulted in a drastic drop in glass transition temperature, while etherified benzylic −OH groups and reductive treatment were observed to increase *T*_g_.

Antioxidant activity is an important property of lignin in various formulations.^54−56^ The radical scavenging index (RSI) reflects lignin ability to scavenge free radicals and it is considered as a benchmark indicator for the antioxidant properties of lignin.^54^ Generally, high antioxidant properties are associated with a high content of PhOH groups,^57^ as PhOH form and stabilize phenoxy radicals by donating H atom (Scheme 1). Even though some works reported that aliphatic −OHs seems to decrease the RSI values because of H-bonding,^58^ others stated that no effect of aliphatic −OH was found in the antioxidant properties.^59^ Thus, the definite assignment of the effect of aliphatic −OHs in RSI is still an open challenge. Herein, we provide further elucidation of the effect of each functionality on the lignin antioxidant properties using the normalized RSI (nRSI) approach (see ESI). The nRSI value for Ind-AT was 8.1 mmol of g^–1^ (Figure 7a). As expected, complete masking of −OH (both aliphatic and phenolics) through acetylation (Ind-Ac) resulted in the (almost) absence of antioxidant activity with an nRSI value of 0.2 mmol g^–1^. Consistently, a positive correlation between nRSI and the number of PhOH groups was found. This finding is in line with the reported mechanism of antioxidant activity using the DPPH free radical method (Scheme 1).^60^ It implies that an increase in PhOH group content promotes the DPPH radical scavenging.

**Scheme 2:**
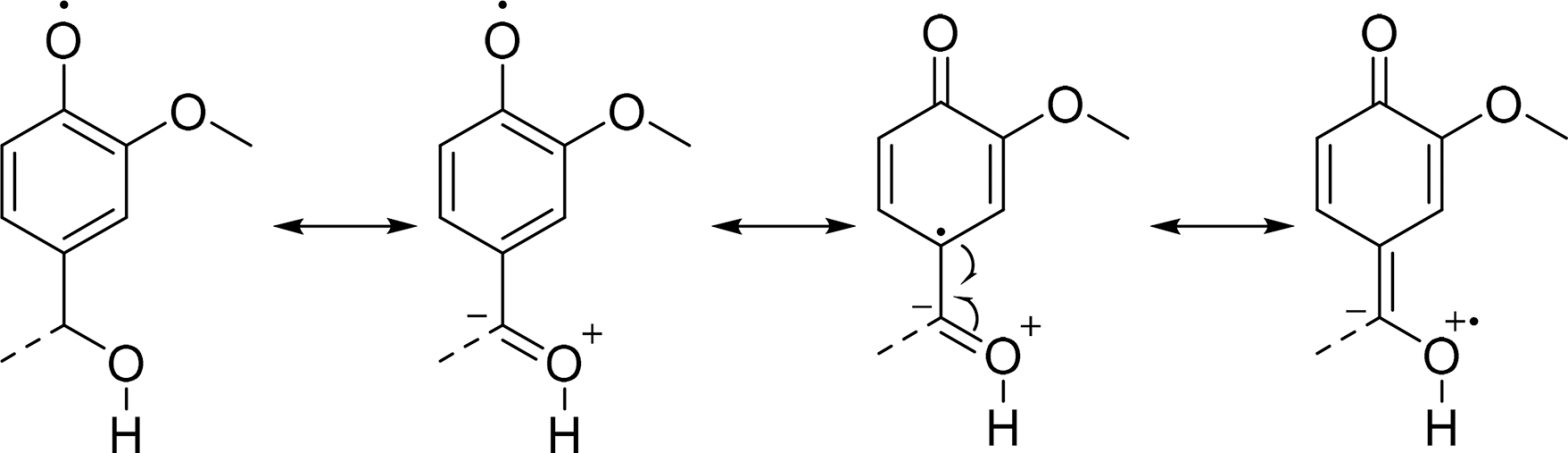
Proposed Mechanism for the Stabilization of Phenoxy Radicals at the Benzylic Position

**Figure 8:**
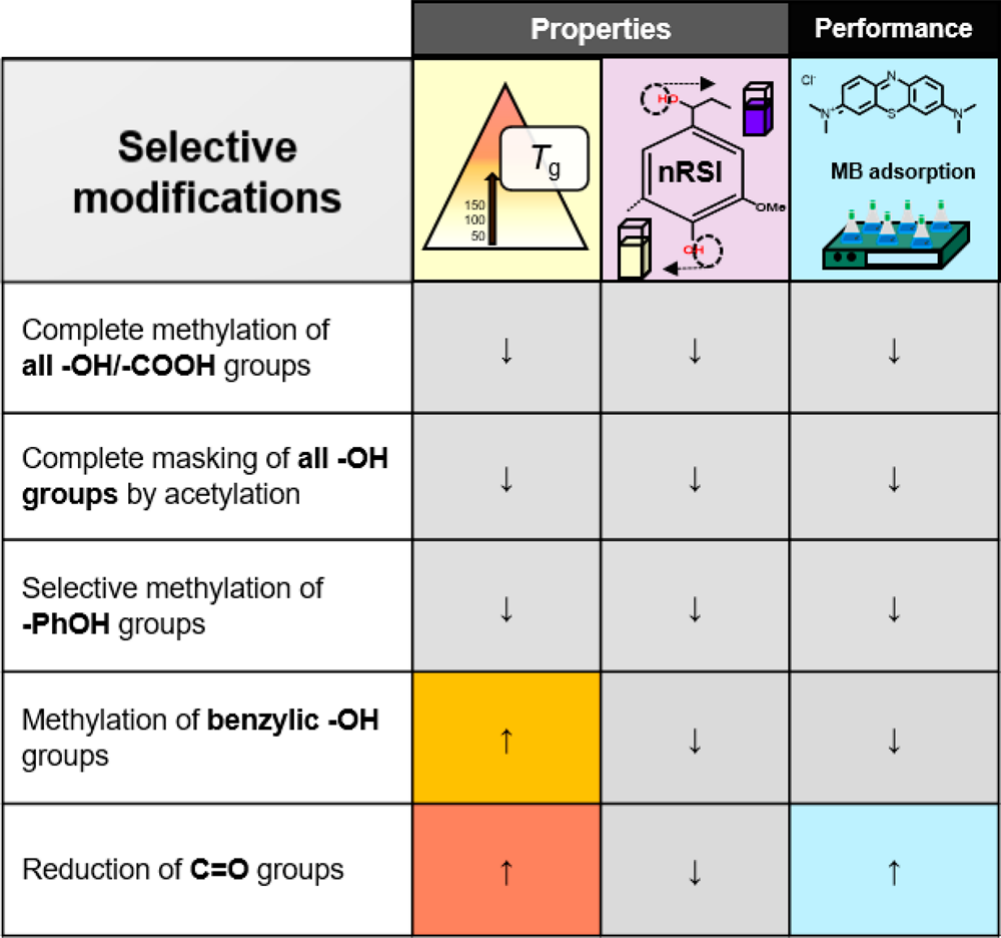
Summary of the effect of different modifications procedures on lignin properties (*T*_g_ and nRSI) and lignin performance (MB adsorption).

Masking PhOH groups in the Ind-Ph sample resulted in a ca. 6-fold decrease of nRSI compared to Ind-AT. The residual scavenging activity in the Ind-Ph sample can be related to the residual PhOH groups (10%; Figure 2, bottom). Methylation of −OH/–COOH groups in Ind-DMS sample resulted in a ca. 4 times lower RSI value compared to Ind-AT (2.1 vs 8.1 mmol g^–1^, respectively), which is consistent with masking phenolic hydroxyl groups. Noteworthy, a ca. 1.5 times higher nRSI value was found for Ind-DMS when compared to Ind-Ph (2.1 vs 1.4 mmol g^–1^, respectively; Figure 7a). Since the amount of the residual PhOH in the latter two samples (Ind-Ph and Ind-DMS) is very close (0.38 mmol g^–1^ and 0.40 mmol g^–1^; see Figure 2, bottom), an effect of aliphatic −OH and −COOH groups cannot be excluded. Hence, Ind-Ph accounts for 15 times higher aliphatic OH content than Ind-DMS (2.12 and 0.14 mmol g^–1^, respectively; see Figure 2, bottom), and this is in line with a detrimental effect of aliphatic −OHs for RSI, due to H-bonding. In addition, an effect of −COOH groups in the formation of H-bonds cannot be excluded as well (0.33 and 0.05 mmol g^–1^ in Ind-Ph and Ind-DMS, respectively; Figure 2, bottom). A similar outcome was achieved for the reduced sample (Ind-R). Ind-R sample with higher aliphatic and phenolic −OH (15% and 8%, respectively) compared to Ind-AT (entries 1 and 6 in Figure 2, bottom) showed lower antioxidant activity (Figure 7a). This result confirms once again that aliphatic −OH is detrimental for the antioxidant properties of lignin. Intriguingly, selective methylation of benzylic −OH groups resulted in a lower scavenging activity with respect to Ind-AT (Figure 8a). Based on 2D NMR and ^31^P NMR results, the formation of β-O-4/α-OMe units and simultaneous decrease by 23% in aliphatic −OH content (Figure 2, bottom; entry 5) lead to the conclusion that masking benzyl-type −OH groups is detrimental for the antioxidant activity of lignin, as the benzylic position might be involved in the stabilization of free radicals (Scheme 2).

The observation that Ind-Me and Ind-R exhibited similar nRSI values (7 mmol g^–1^; Figures 2 and 7a) is difficult to explain, since the two samples possess different aliphatic and phenolic −OH and −COOH group content (they differ for more than one parameter).

#### Thermal Properties

Selective masking of lignin functionalities allowed us to detect the changes in the thermal behavior of the modified samples. Glass transition temperatures (*T*_g_) of the modified lignins fit the range 105–185 °C (Figure 7b). Earlier reported data demonstrated a gradual decrease in *T*_g_ as the degree of substitution increases.^61−63^ A 35% decrease in *T*_g_ was indicated for the acetylated lignin compared to Ind-AT due to the increased chain mobility and plasticizing effect of the acetic groups. In contrast, complete methylation did not result in any change in *T*_g_, which might be attributed to the added stiffness (lower statistical degrees of freedom) to the lignin chains by incorporation of stable −OCH_3_ groups. Unexpectedly, selective methylation of β-O-4/α-OH groups contributed to the 11% increase in *T*_g_ comparing to Ind-AT (Figure 7b). To explain this, effects such as configurational stability, steric hindrance, and other low-energy interactions (i.e., van der Waals forces) should be considered in addition to the mere plasticizing effects of the substituents. *T*_g_ of Ind-Ph sample dropped from 158 to 134 °C, which is in good agreement with the results obtained by Cui et al. for the selectively PhOH methylated lignin.^64^ Elimination of most hydrogen bonds through the masking of PhOH groups leads to the expected decrease in *T*_g_. Interestingly, the glass transition point of the reduced lignin showed a drastic jump from 158 °C in the reference Ind-AT to 185 °C in the reduced lignin (Ind-R). This is in line with an increased number of H-bonds together with a higher molar mass distribution (Figures 7b and 4 and Table 3).

## Conclusions and Future Perspectives

We here report a study toward a more reliable approach to unveil lignin structure–property–performance correlation by selectively masking one specific lignin functionality at a time, followed by a properties and performance evaluation. Methylene blue adsorption was chosen as a fast and small-scale screening method to evaluate the performance behavior of the modified (masked) lignins, while glass transition temperature (*T*_g_) and antioxidant activity (nRSI) were selected as the properties. Figure 8 gives a comprehensive overview of the effect of each modification on the measured properties and performance.

To summarize, phenolic and aliphatic −OH groups (total) are almost equally important in improving the lignin sorption capacity. Noteworthy, our results show for the first time that benzylic −OH plays a preeminent role with an effective contribution ca. 3 times higher than the one of phenolic −OH.

Masking benzylic −OH groups and reduction led to an increase in *T*_g_ values by 11% and 17%, respectively (Figure 7b), while masking all aliphatic and phenolic −OH groups resulted in a lower glass transition temperature. nRSI experiments revealed a decline in the antioxidant properties for all modified lignins with respect to Ind-AT (Figure 7a), meaning that the modifications performed are not suitable to improve the antioxidant activity of lignin. As expected, we found that PhOH play a crucial role in increasing the antioxidant activity of lignin, while aliphatic −OH groups and −COOHs are detrimental to nRSI, most likely due to H-bonding.

The proposed approach bridges the gap toward efficient lignin engineering by estimating the suitability of each specific lignin for a particular application. Though few properties were investigated, in our vision, future studies should implement similar approaches when comparing the properties and performance of different lignin samples. The currently existing bottleneck implies the absence of a fast-track screening method and, thus, the inability to establish solid structure–property–performance correlation. In contrast, our approach can be used as a first step in lignin-based product development, since it allows one to gain insights into the lignin behavior in selected properties and applications.

However, the presented approach possesses major open challenges that should be improved. Examples include expanding the library of studied samples together with considering other properties and applications for a high-throughput and more comprehensive lignin engineering. In addition, further optimization of the modification procedures provides an opportunity to accelerate the lab work and make the approach easier “to be handled”. In that regard, a library of standardized protocols should be implemented. In addition, for certain applications (i.e., materials performance), the scale-up of the analytical modification is of key importance.

Overall, this new approach is a step further toward the development of an efficient, reliable, and effective tool for lignin engineering. Nevertheless, further studies, maybe complemented with AI-modeling, are needed to provide a generally applicable methodology.

## Supporting Information Available

Details about the molar mass distribution (MMD), the thermogravimetry analysis (TGA), the antioxidant activity, and the DSC curves of the modified samples (PDF)

## Dedication

This work is dedicated to Professor of Practice Dr. Mikhail Balakshin.
