# Supplementary material for: Study toward a More Reliable Approach to Elucidate the Lignin Structure–Property–Performance Correlation
Source: Biomacromolecules. 2023 Dec 19;25(1):200–12. doi: 10.1021/acs.biomac.3c00906 (PMC10777350; doi:10.1021/acs.biomac.3c00906)
Supplement: Supplementary file 1 — bm3c00906_si_001.pdf [file bm3c00906_si_001.pdf]

Electronic Supporting Information (ESI)

A study towards a more reliable approach to elucidate lignin structure-properties-performance correlation

Daryna Diment[a], Oleg Tkachenko[b], Philipp Schlee[a], Nadine Kohlhuber[c], Antje Potthast[c], Tetyana M. Budnyak[b]*, Davide Rigo[a]* and Mikhail Balakshin[a]†

[a]*Department of Bioproducts and Biosystems, School of Chemical Engineering, Aalto University, 02150, Espoo, Finland*

[b]*Division of Nanotechnology and Functional Materials, Department of Materials Science and Engineering, Uppsala University, 751 03, Uppsala, Sweden*

[c]*Institute of Chemistry of Renewable Resources, Department of Chemistry, University of Natural Resources and Life Sciences (BOKU), 3430, Tulln, Austria*

† Deceased

*Email: davide.rigo@unive.it

tetyana.budnyak@angstrom.uu.se

In memoriam

Professor of Practice Dr. Mikhail Balakshin († 2022)

Table of contents:

Molar mass distribution (MMD) of the modified samples……………………………………………………S2
Thermo gravimetry analysis (TGA) of the modified samples…………………………………………………...S3
Antioxidant activity of the modified samples…..………………………………………………………..…S4
DSC curves of the modified samples………..………………………………..………………………………S5

Molar mass distribution (MMD) of the modified samples

Table S1. Statistical moments of Ind-AT and after blank reduction

	M_n (Da)	M_w (Da)	M_z (Da)	Đ
Ind-AT	1600	10400	60500	6.49
Ind-R	3800	17600	75100	4.68

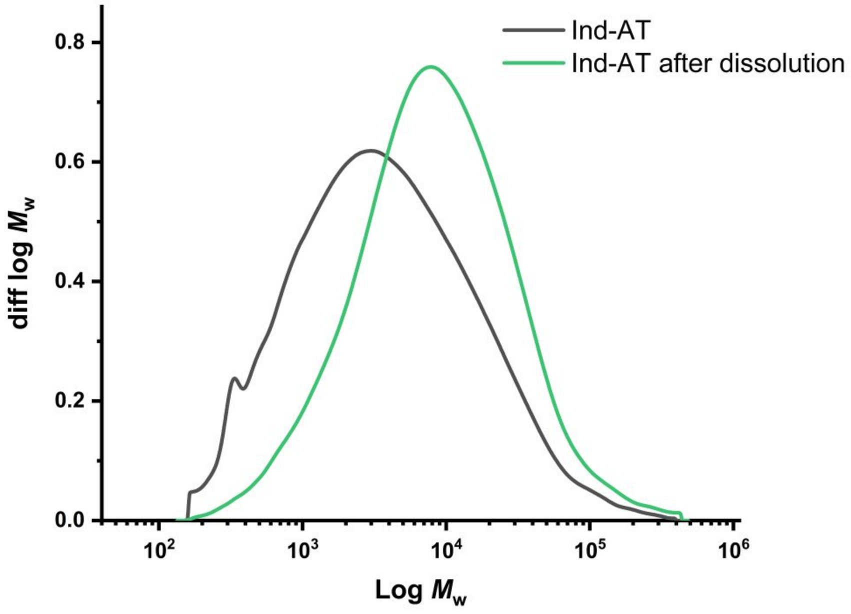

Figure S1. Molar mass distributions of Ind-AT before and after blank reduction

Thermo gravimetry analysis (TGA) of the modified samples

Ash content was determined by using Netzsch STA 449 F3 Jupiter & QMS 403 Aëolos Quadro thermal analyzer. The TGA procedure was performed by heating the samples (ca. 10-15 mg) from 40 °C to 600 °C under air (50 mL min^{-1}) and nitrogen (20 mL min^{-1}) atmosphere at a heating rate of 10 °C min^{-1}. Ash content was precisely defined using Proteus® software for thermal analyzers.

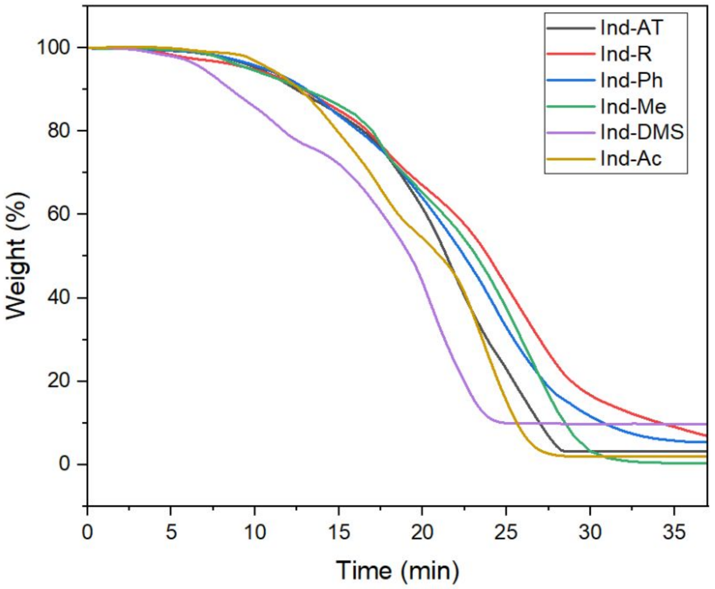

Figure S2. TGA of the modified lignins and Ind-AT (reference)

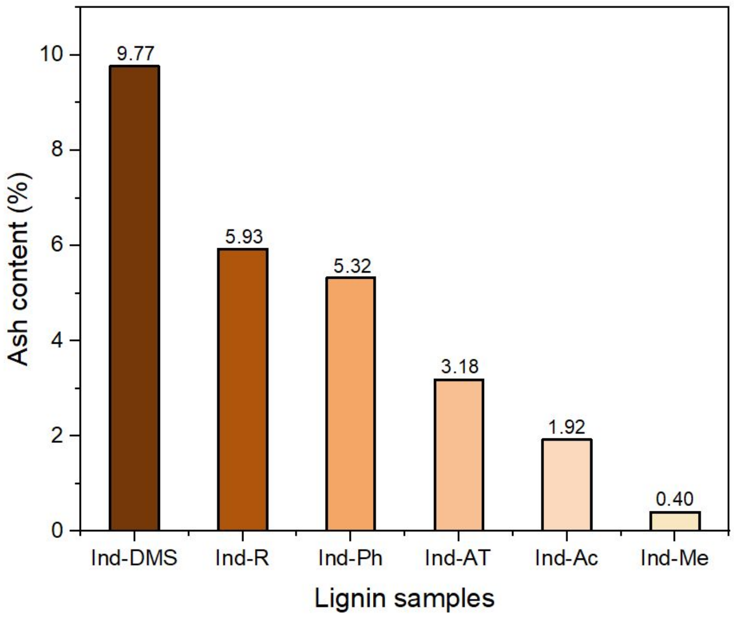

Figure S3. Ash content of the investigated samples.

Antioxidant activity of the modified samples

Radical scavenging index (RSI) was used to reflect the antioxidant properties of lignins. The percentage of the scavenged DPPH radicals was reflected in the form of inhibition percentage (IP). IP values were corrected due to the DPPH degradation by adding to the original absorbance values the difference between the absorption at 0 and the corresponding time point of the blank solution (24 h) which consists of DPPH solution and lignin solvent without adding lignin itself calculated by equation 1. Effective concentration (EC_{50}) of lignin at which 50 % of DPPH were neutralized, was found by plotting IP as a function of the real lignin concentration in the lignin-DPPH solution (3-15 mg L^{-1}) (Figure S4a). Following that, the data points were fitted with polynomial equations and the EC_{50} was found as shown in Figure S4b. The latter is obtained considering the real mass of lignin correction and the dilution occurring when mixing the solutions. To avoid erroneous estimation of the lignin antioxidant properties, EC_{50} value in mg L^{-1} was normalized by DPPH concentration in mmol L^{-1} (nEC_{50}) (Equation 2). Following that, inversed value of nEC_{50} was defined as nRSI in mmol g^{-1} which stands for mmol of DPPH which can be scavenged by 1 g of lignin in 24 h (Equation 3).

$$\mathrm{IP}(\%) = \frac{\mathrm{A_0}(\mathrm{absorbance}_{t=0}) - (\mathrm{A_t}(\mathrm{absorbance_t}) + (\mathrm{A_{0DPPH}} - \mathrm{A_{tDPPH}}))}{\mathrm{A_0}(\mathrm{absorbance}_{t=0})} \times 100 \qquad (Eq.1)$$

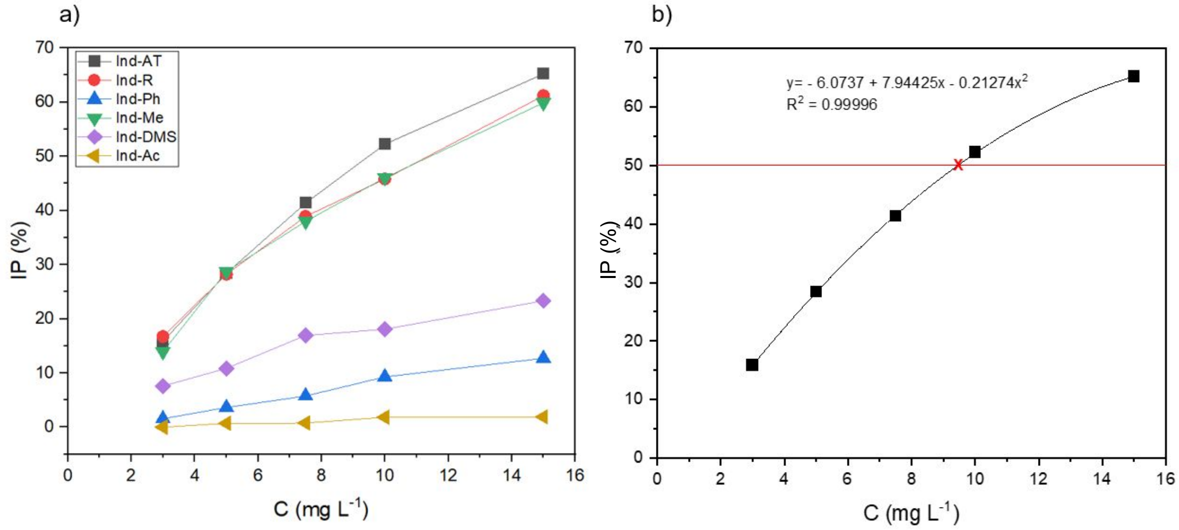

Figure S4. a) IP values (%) of the modified lignins and Ind-AT after 24 h as a function of the real concentrations (3-15 mg L^{-1}) of the given lignins in the lignin:DPPH solutions; b) IP values of Ind-AT were found to fit 2nd order polynomial equation. EC_{50} was found by resolving the equation and indicated as **x** on the plot.

$$\mathrm{nEC_{50}} = \frac{\mathrm{EC_{50}}}{[\mathrm{DPPH}]} \qquad (2)$$

$$\mathrm{nRSI} = \frac{1}{\mathrm{EC_{50}}} \qquad (3)$$

DSC curves of the modified samples

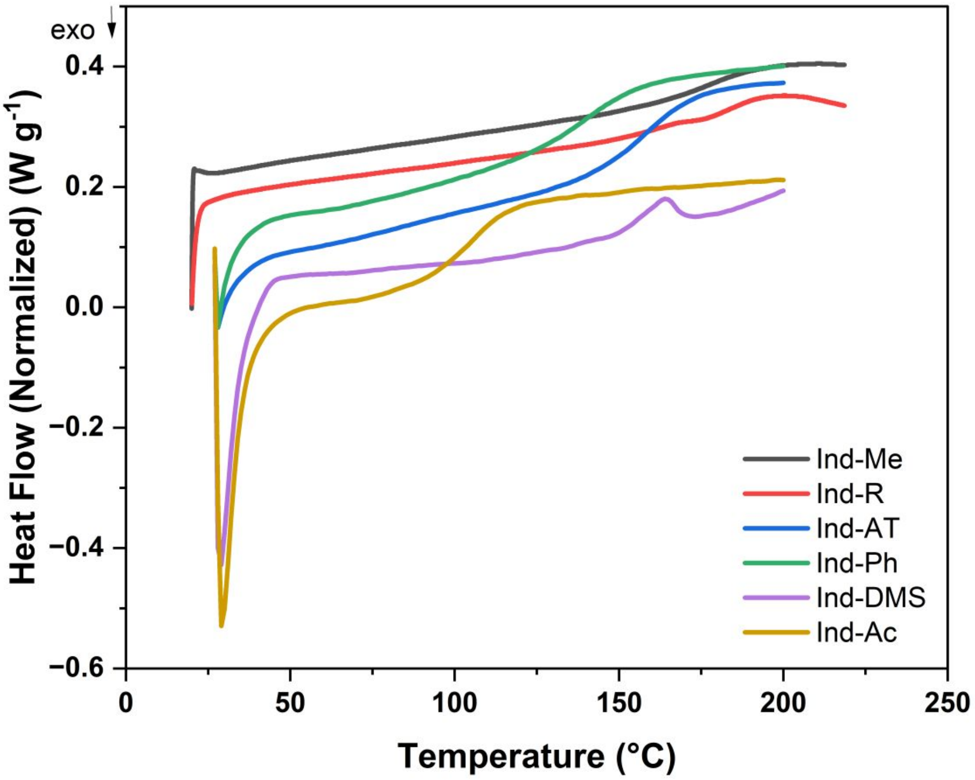

Figure S5. DSC curves of the modified lignins and Ind-AT (reference)
